# Source Attribution of the Chemical Warfare Agent Soman Using Position-Specific Isotope Analysis by ^2^H NMR Spectroscopy: From Precursor to Degradation Product

**DOI:** 10.1021/acs.analchem.1c01271

**Published:** 2021-09-01

**Authors:** Sandra Lindberg, Magnus Engqvist, Lina Mörén, Crister Åstot, Rikard Norlin

**Affiliations:** Department of CBRN Defence & Security, The Swedish Defence Research Agency (FOI), Cementvägen 20, Umeå SE-901 82, Sweden

## Abstract

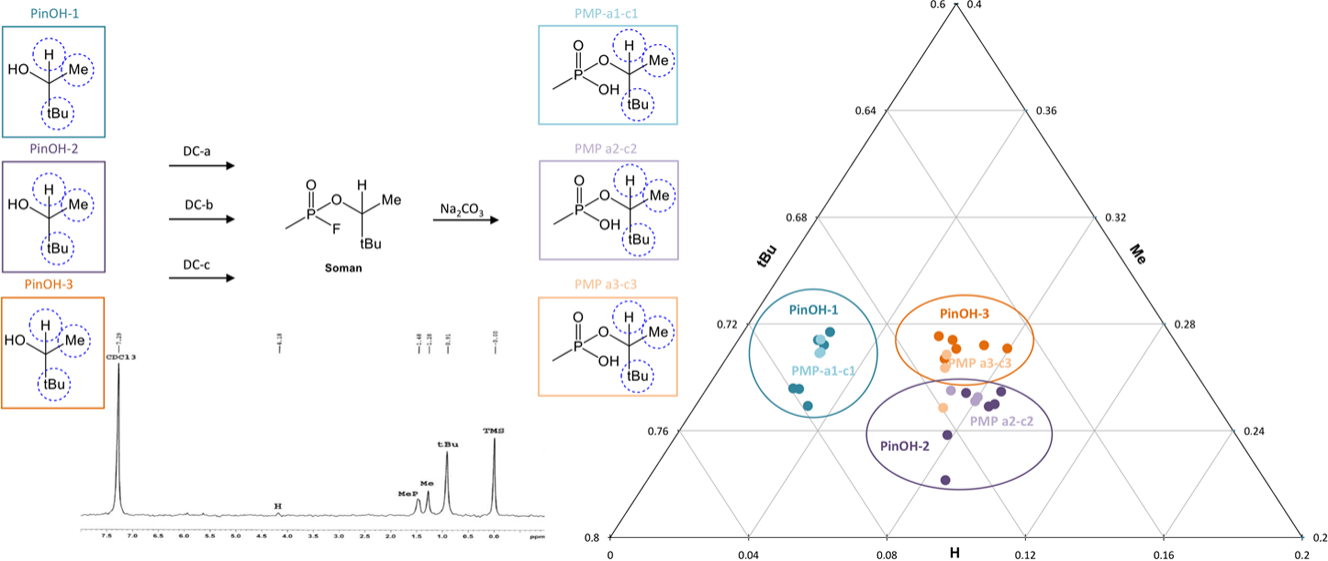

Position-specific isotope analysis (PSIA) by NMR spectroscopy is a technique that provides quantitative isotopic values for every site—a so-called isotopic fingerprint—of a compound of interest. The isotopic fingerprint can be used to link samples with a common origin or to attribute a synthetic chemical to its precursor source. Despite PSIA by NMR being a powerful tool in chemical forensics, it has not yet been applied on chemical warfare agents (CWAs). In this study, different batches of the CWA Soman were synthesized from three distinctive pinacolyl alcohols (PinOHs). Prior to NMR analysis, the Soman samples were hydrolyzed to the less toxic pinacolyl methylphosphonate (PMP), which is a common degradation product. The PinOHs and PMPs were applied to PSIA by ^2^H NMR experiments to measure the isotopic distribution of naturally abundant ^2^H within the pinacolyl moiety. By normalizing the ^2^H NMR peak areas, we show that the different PinOHs have unique intramolecular isotopic distributions. This normalization method makes the study independent of references and sample concentration. We also demonstrate, for the first time, that the isotopic fingerprint retrieved from PSIA by NMR remains stable during the production and degradation of the CWA. By comparing the intramolecular isotopic profiles of the precursor PinOH with the degradation product PMP, it is possible to attribute them to each other.

The character of investigations into alleged chemical weapons use has shifted sharply in recent years. Previously, the chemical analysis of chemical warfare agents (CWAs) in environmental materials sampled at the scene was sufficient to provide evidence that a toxic chemical or its degradation products was present at the site. The Chemical Weapons Convention (CWC), the Organization for the Prohibition of Chemical Weapons (OPCW) and its verification regime were established for armed conflicts of the past. With the changing circumstances of modern conflicts with no clear adversaries, fractionalized battlefields and a more complex situation with civilians in the war zones, other demands are starting to emerge for the analysis of CWA. Questions that now require answers are not only if a CWA has been used but also where-, how- and with which starting materials was the CWA produced? In recent years, CWAs have been used for chemical attacks in the Syrian Arab Republic, Malaysia, the United Kingdom, and the Russian Federation. In the investigations of such incidents, these new types of forensic questions have to be addressed. In the case of the Syrian Arab Republic, OPCW has deployed a number of fact-finding missions (FFMs)^1^ to provide clarity on the circumstances of specific incidents. In the Malaysia and the United Kingdom cases, it has been the responsibility of the public law enforcement authorities to conduct the investigations.^2,3^ In all of these cases, the identification of the actual CWA has only confirmed the severity of the incident, and more information from crime scene samples has been requested. Such an approach to sampling and analysis resembles that of classic forensic drug investigations and sets different and higher demands on the chemical analysis of samples. The recent development has also encouraged OPCW to announce a temporary working group (TWG)^4^ on investigative science and technology and to establish an investigation and identification team (IIT).^5^ IIT has the mandate to attribute the use of CWA and identify the perpetrators of chemical weapons use in the Syrian Arab Republic. In this context, the support of the scientific community through research into more precise chemical analysis for attribution of CWA samples is much needed.

Chemical profiling of samples during forensic investigations can generate information to create a link between samples and/or indicate their origin (i.e. source attribution). The chemical attribution signatures (CAS) analyzed can be classified into (i) extrinsic CAS, such as impurities or additives (organic and inorganic), and (ii) intrinsic CAS, such as stable isotope profiles. In the case of extrinsic CAS, there is a need for reference data to be able to link the suspect sample to a synthesis route, specific starting material, technical competence of the perpetrator, etc. Methods for route sourcing have been developed for the CWAs Russian VX^6^ and sulfur mustard.^7^ Additionally, source attribution using extrinsic CAS such as trace contaminants present in CWA precursors and products has been developed. An example is the link between the nerve agent precursor DC and the synthesis product Sarin.^8^ The integrity of intrinsic CAS as isotope profiles of natural abundance can be relatively conserved from precursors to products in a chemical reaction, although minor changes might take place due to the isotope fractionation.^9,10^ Position-specific isotope analysis (PSIA) by NMR spectroscopy can provide isotopic information of individual positions within a molecule, compared to measurements by isotope ratio mass spectrometry (IRMS) that provide a global isotope composition. The site specificity achieved with PSIA by NMR will substantially increase the number of parameters and therefore the resolution of the isotopic information since a variation between different sites often can be expected. Thus, PSIA by NMR has proven to be useful in a wide range of scientific applications, for instance, in food chemistry, to ensure the authenticity of natural products,^11,12^ pharmaceutical patent infringement,^13,14^ tracking of illicit drug distribution,^15,16^ and to reveal drugs’ geographical origin.^17^ Intrinsic CAS, such as naturally abundant δ^13^C in the CWA precursor methylphosphonic dichloride (DC) and its degradation products by GC-IRMS, have previously been published.^18^ However, to our knowledge, investigations on the pathway of precursor to a degradation product of a CWA with PSIA by NMR have not yet been published.

The intramolecular distribution of ^2^H in organic molecules can show significant variations depending on the synthetic origin of the molecule. The ^2^H NMR signal integrals are ideally directly proportional to the number of detected ^2^H nuclei, and therefore, to the isotope abundance.^19^ In this study, we have acquired quantitative ^2^H NMR data to monitor the isotopic distribution of natural abundance ^2^H of the Soman precursor pinacolyl alcohol (PinOH) and the degradation product pinacolyl methylphosphonate (PMP). Furthermore, we have investigated if the intramolecular isotopic profile of the pinacolyl moiety remains through the synthesis and hydrolysis by exploring the possibility to attribute PinOH to PMP, and vice versa, based on their isotopic profiles.

## Experimental Procedures

### Safety

All nerve agents, Soman included, are highly toxic compounds. Appropriate protective measures must be taken to ensure that neither the personnel nor the environment comes in contact with these chemicals. All labware that has come in contact with Soman must be thoroughly decontaminated (e.g., alkaline alcohol) after use and the waste disposed properly. The synthesis of CWA is restricted by international agreements (CWC) and national legislation, and if the appropriate authorization is not in place, the work will be considered criminal.

### Chemicals

Pinacolyl alcohols (3,3-dimethyl-2-butanol, CAS No. 464-07-3) were received from PinOH-1 (unspecified purity, Kebo Düsseldorf, Germany); PinOH-2 (98%, Sigma Aldrich, St. Louis, MI) and PinOH-3 (99%, Alfa Aesar, Haverhill, MA). DC (Methylphosphonic dichloride, CAS No. 676-97-1) were received from: DC-a (98%, Alfa Aesar, Haverhill, MA); DC-b and DC-c were both synthesized in-house using established methods.^20^ Other chemicals used in the synthesis were commercial and used as received without further purification.

The chloroform used in the NMR samples, CDCl_3_ (100% D, CAS No. 865-49-6) and CHCl_3_ (99–99.6%, CAS No. 67-66-3), were purchased from Glaser Labkemi (Gothenburg, Sweden) and VWR (Radnor, PA), respectively.

### Synthesis of Soman and Pinacolyl Methylphosphonate (PMP)

Nine unique batches of PMP (PMP-a1-c3) were achieved via hydrolysis of Soman, which was synthesized using different combinations of the starting materials DC and PinOH: three diverse DCs (DC-a, DC-b, and DC-c) and three PinOHs (PinOH-1, PinOH-2, and PinOH-3). The ^2^H diversity within the DCs is not included in this study. The solvent was evaporated, and the nine batches of Soman were used without further purification. All Soman batches were then converted into the hydrolysis products PMP by treatment with a 10% solution of Na_2_CO_3_ (aq.). After hydrolysis, the nine samples were extracted with CHCl_3_, dried with Na_2_SO_4_, and evaporated, yielding PMPs with purities all above 94% according to ^1^H- and ^31^P NMR.

### Sample Preparation Prior to ^2^H NMR Analysis

Quantitative ^2^H NMR experiments were performed on samples of PMP in chloroform (CHCl_3_). The amount of PMP was weighed in vials and dissolved in appropriate amounts of chloroform (CHCl_3_) to achieve stock solutions with the same concentrations (1.78 M). Then, 400 μL of the stock solution was transformed to a NMR tube and further diluted (CHCl_3_) to give a final volume of 550 μL. The resulting NMR samples had concentrations of 1.29 M, limited by the synthesis batch that had produced the smallest amount of the product, 128 mg (0.71 mmol) in 550 μL of CHCl_3_. Quantitative ^2^H NMR experiments were applied to both neat and diluted samples of PinOH. The neat samples were prepared without any solvent, while the diluted samples were measured by volume, then diluted in CHCl_3_ to achieve the same concentration of the stock solution as for the samples of PMP. Then, as for PMP, 400 μL of the stock solution was transformed to a NMR tube and further diluted (CHCl_3_) to a total volume of 550 μL. All stock solutions and NMR tube dilutions were temperature-stabilized, prepared, sealed, and gently shaken just prior to the first ^2^H NMR experiment. Afterward, the NMR samples and vials with stock solutions were covered with parafilm and stored in a refrigerator to prevent evaporation of the solvent before the replicate measurements were performed.

### ^2^H NMR Analysis

The quantitative ^2^H experiments were achieved on a Bruker Avance 500 MHz spectrometer equipped with a 5 mm broadband cryogenic probe head. The measurements were performed under composite pulse decoupling (WALTZ-16) conditions and at a temperature of 298 K. All signals in the ^2^H NMR spectra of PMP were assigned from the corresponding ^1^H NMR spectra having signals with very similar chemical shifts; (CDCl_3_, δ, ppm): 9.31–9.11 (1H, s, OH), 4.23–4.16 (1H, dq, *J* = 8.9 and 6.4 Hz, CH), 1.52–1.45 (3H, d, *J* = 18.0 Hz, PCH_3_), 1.31–1.27 (3H, d, *J* = 6.4 Hz, CH_3_), 0.92 (9H, s, tBu). Correspondingly, the ^1^H NMR signals for PinOH are (CDCl_3_, δ, ppm): 3.52–3.43 (1H, dq, *J* = 6.4 and 5.0 Hz, CH), 1.53–1.51 (1H, d, *J* = 5.0 Hz, OH) 1.13–1.10 (3H, d, *J* = 6.4 Hz, CH_3_), 0.89 (9H, s, tBu). All ^2^H NMR experiments were routed through the lock channel and, as a consequence, the experiments were performed unlocked. The probe was matched and tuned on a reference NMR sample (550 μL CDCl_3_) to the recording frequency of 76.77 MHz and then manually shimmed. After shimming, the reference sample was replaced with the sample to be analyzed by the ^2^H NMR experiment, containing PinOH or PMP in CHCl_3_. This sample replacement procedure was applied to all ^2^H NMR experiments. A minimum of three independent spectra were recorded for each PinOH and for each PMP, collecting at least 4096 scans (13 h 44 min) for every experiment. The replicates were performed in different manners: some were recorded in a sequence; some in intervals with new tuning and shimming on the reference sample with CDCl_3_ in between; and, when the amount of sample made it possible, some replicates were recorded on different aliquots (400 μL) from the same stock solution. The acquisition time was 9.99 s and the spectral width 1535 Hz. The time domain was 30k and zero-filler twice to 65k. To ensure total relaxation of the magnetization for all deuterium nuclei, the relaxation delay was set to 2.0 s. Due to the lack of precision and sensitivity in the ^2^H relaxation time (*T*_1_) measurements, the *T*_1_ of the corresponding protons were determined (2.0–4.7 s). According to literature, the deuterium nuclei could have shorter relaxation times by a factor of 10 or more, compared to protons at equivalent position.^21^ The free induction decay (FID) was submitted to an exponential multiplication inducing a line broadening of 2.0 Hz. After automatic phasing and baseline correction with an adequate polynomial,^11^ the signal-to-noise ratio (SNR) of all ^2^H peaks of the PinOHs and PMP are equal to or greater than ten (SNR ≥ 10).

### Processing of ^2^H NMR Data

**Figure 1:**
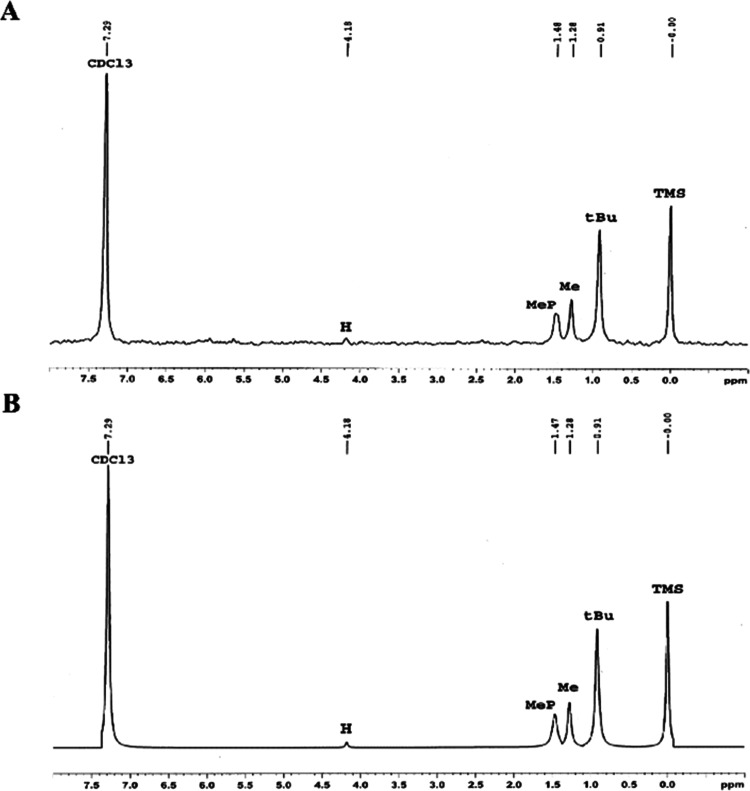
(A) Example of a quantitative ^2^H NMR experiment of PMP-a3, showing the pinacolyl signals for position **H**, **Me**, and **tBu**. The less intense signal **H** has a SNR ≥ 10. The **CDCl**_**3**_, **MeP**, and **TMS** signals were not considered in this study. (B) Corresponding peak signal curve fitted using a 100% Lorentzian line shape.

All peaks were manually picked. By processing the ^2^H NMR spectra with the Bruker TopSpin (3.5) deconvolution application DCON, all peak areas were determined using a 100% Lorentzian line shape. Three peak areas were retrieved from the pinacolyl moiety, representing the position of the single ^2^H (**H**), the methyl group (**Me**), and the *tert*-butyl group (**tBu**) containing the possibility of one, three, and nine equivalent deuterium nuclei, respectively (Figure 1). Examples of the experimental, simulated data and the difference between them (residual), and also the fitted shapes of the deconvolution, appear in the Supporting Information.

Each sample’s peak areas were normalized such that the total area for all tree peaks equals one. The normalized ^2^H mean values of all replicates were then calculated for every position of the pinacolyl moiety in the PinOHs and in the PMPs, respectively (Table S1).

## Results and Discussion

**Figure 2:**
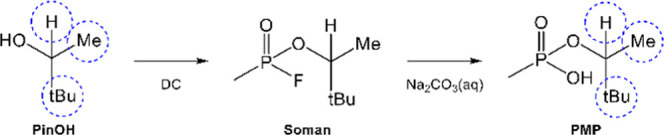
Synthesis route of unique batches of Soman and its hydrolyzed product PMP (PMP-a1-c3) through the “di-di” method, using diverse PinOHs (PinOH-1-3) and DC (DC-a-c). The figure is highlighting the positions (**H**, **Me**, and **tBu**) within the pinacolyl moiety explored by ^2^H NMR experiment.

Nine unique batches of the organophosphorus nerve agent Soman were produced using different combinations of precursors (Figure 2). The three PinOHs and three DCs had different origins; some were commercial from different suppliers, and some were produced in-house to ensure their diversity. In CWA forensics, suspect illicit use of Soman is expected to use PinOH in the final step of the production, and it would be the primary task of laboratories to correlate that PinOH to the Soman or to the degradation product. Because of that, further background of the commercial PinOHs, such as subcontractors, country of origin, and method of production (synthesis or extraction from a natural source) were not investigated as part of this study.

Since this study was exploratory and the pinacolyl moiety holds a sufficient number of parameters (three individual positions) to make an intramolecular isotopic profile, we did not use any certified internal standard with a known ^2^H/^1^H ratio (or working standard calibrated against the certified standard) to get an absolute value for every position. Nor do we know the whole molecules average δ^2^H provided by IRMS. Instead, each sample’s peak areas were normalized to get an intramolecular ^2^H profile. The normalization method holds an advantage in being stand-alone without any need for reference or another analysis technique. However, the relative values received within this study can only be used when comparing isotope profiles between samples of the same scaffold measured either in the starting materials or in the products.

**Figure 3:**
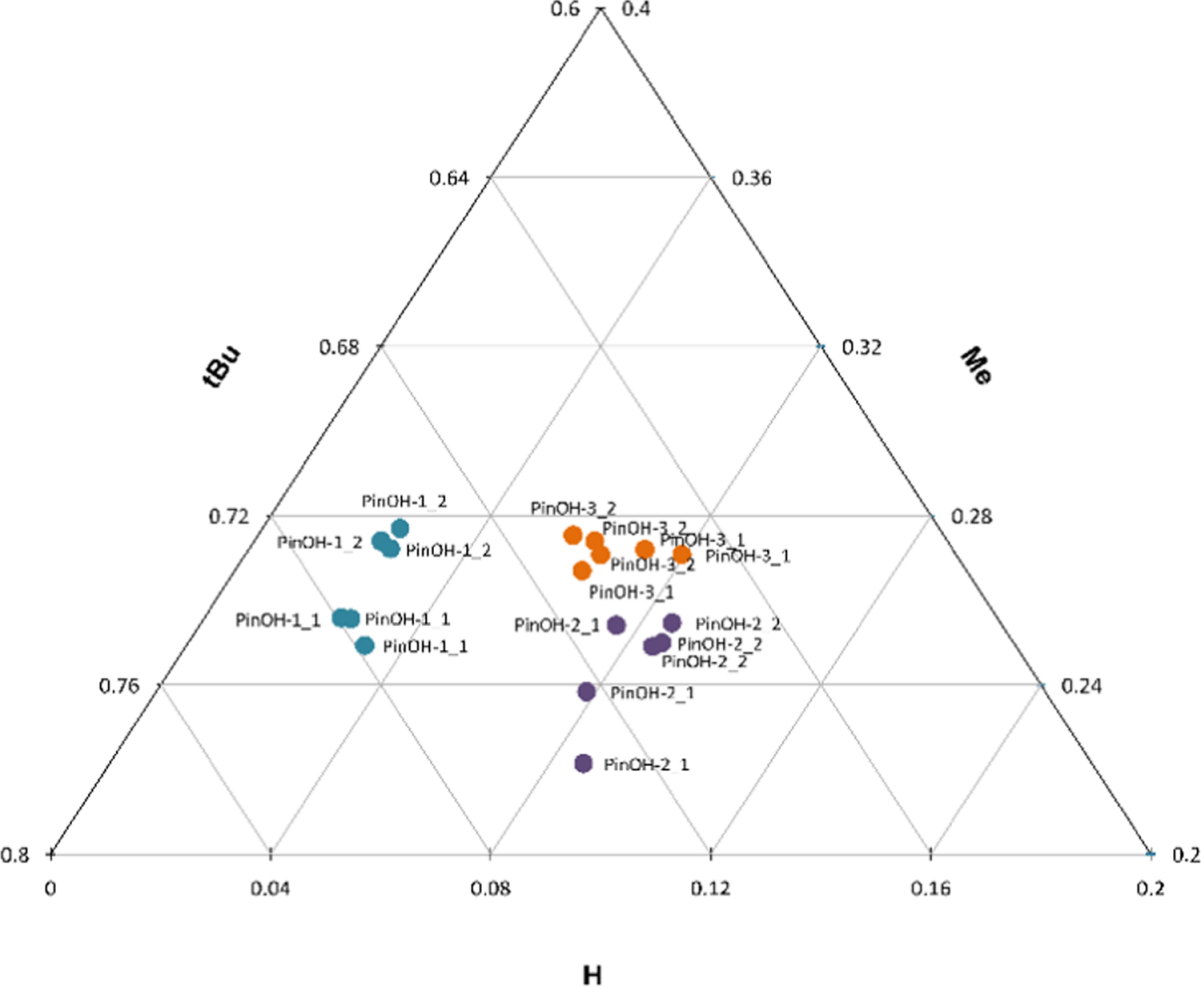
Ternary plot with the pinacolyl positions **H** versus **Me** versus **tBu**, showing all normalized ^2^H peak area replicates for the precursors (PinOH-1-3_1 (dil.) and PinOH-1-3_2 (neat)). The sum of the ^2^H peak areas for all three positions equals one.

Initially, the resolving power of the NMR method was investigated by the analysis of the three diverse PinOHs (PinOH-1-3). The intramolecular ^2^H profile within the alkyl chain differed, and the three PinOHs had unique profiles. The isotopic profiles are visualized in a ternary plot in which the axis represents each position of the pinacolyl moiety (**H** versus **Me** versus **tBu**) (Figure 3). The sum of the ^2^H peak areas for all positions equals one.

The normalized peak areas were collected from a triplicate of the two different sample concentrations. Since this study focuses on the intramolecular isotopic pattern, the analysis will be independent of sample concentration. Concentration independence is an advantage when analyzing authentic samples as long as there is an adequate amount of sample to get a satisfactory SNR. Additionally, our method is independent of sample purity as long as there are no interferences such as signal overlapping of impurities.

The replicates of each precursor PinOH (PinOH-1-3) cluster together and result in three group formations. PinOH-1 has a unique profile with regard to the ^2^H abundance in the **H**-position, differentiating PinOH-1 from the other two precursors (PinOH-2 and PinOH-3) in this dimension. PinOH-2 and PinOH-3, not as differentiated at the **H**-position, are instead mainly separated in the **Me**-position.

**Figure 4:**
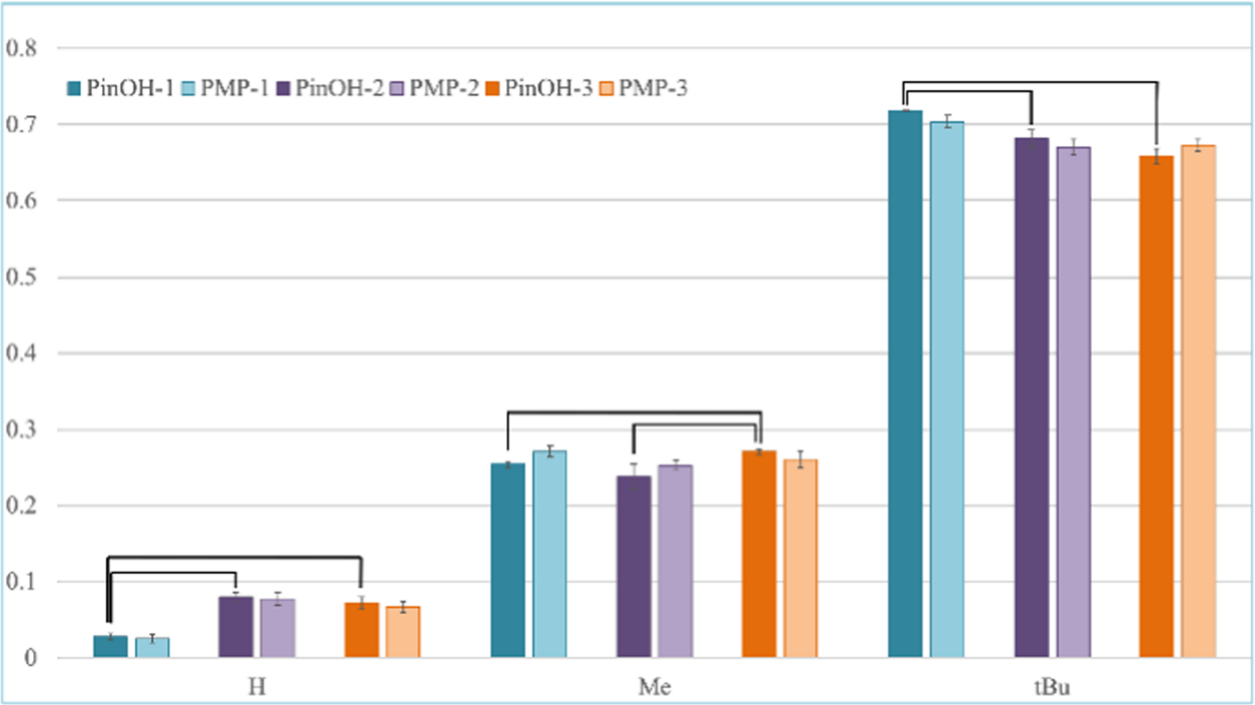
Normalized ^2^H peak areas for the pinacolyl position **H**, **Me**, and **tBu**, showing the correlation in ^2^H abundance between the starting material PinOH (PinOH-1-3, dark left bars, *n* = 6) and the product PMP (PMP-1-3, light right bars, *n* = 9). The error bars show the calculated standard deviations. Statistically significant sample means, with an α level of 0.05, are marked in the graph.

To investigate the statistical significance in the data, the mean value of the normalized peak area for all three positions **H**, **Me**, and **tBu** of PinOHs were compared using a Student’s *t*-test with an α level of 0.05. Statistically significant sample means are highlighted in Figure 4 and two of the three PinOHs have statistically differentiated isotopic abundant ^2^H at the different positions (**H**, **Me**, **tBu**). The majority of the variances are significant. The insignificant differences in position **H** and **tBu** between PinOH-2 and PinOH-3 could be due to differences in ^2^H abundance under our detection limit. However, in accordance with Figure 3, PinOH-2 and PinOH-3 are significantly differentiated in the **Me**-dimension.

Second, we examined if the PinOHs’ unique isotope signatures were stable through the reaction steps by which Soman was first synthesized and subsequently hydrolyzed to PMP. Due to its stability,^22^ PMP is a common degradation product of Soman found in both environmental and biomedical samples and is therefore likely to be discovered in, for instance, soil samples long after Soman has been dispersed. In theory, the pinacolyl moiety should not be directly involved nor affected by any major isotopic fractionation in the chemical reactions from precursor to end product. Therefore, the isotopic distribution of naturally abundant ^2^H within the pinacolyl moiety of the starting material should remain intact and be reflected in the product.

^2^H NMR experiments were recorded for every PMP. The data suggests that there are clear differences in the ^2^H abundance between the products and that the position-specific isotopic information is largely stable through the transformation from the precursor. For comparison, the mean values of the normalized peak area for the precursor PinOHs together with the PMPs are visualized in a bar chart showing every position of the pinacolyl moiety: the single ^2^H (**H**), the methyl group (**Me**), and the *tert*-butyl group (**tBu**) (Figure 4). In the chart, the darker colored left bars represent data from the starting materials, PinOH-1-3, collected from triplicate measurements of each alcohol at different concentrations (*n* = 6). The lighter colored right bars represent data from the corresponding product PMPs (PMP-1-3) deriving from three PMPs (i.e., PMP-1 = PMP-a1, PMP-b1, and PMP-c1 originating from PinOH-1 and different DCs) with three replicates measured for each product (*n* = 9).

In the data, the highest relative standard deviation was recorded for PinOH-1 and PMP-1 at position **H** (10.5%). Since this position only contains the possibility of one equivalent nucleus, it is producing a NMR signal with relatively low intensity. Therefore, the position is vulnerable to noise and, as a consequence, has the lowest signal-to-noise ratios (SNR ≥ 10). Additionally, the data shows that both PinOH-1 and PMP-1 have relatively low natural abundances of ^2^H in this specific position, which is reflected by a further reduction in NMR signal intensities. Because the SNR has a direct impact on the measurement precision,^23^ a low value is more likely to give an inaccurate reproducibility. Therefore, one can expect and accept a relatively high standard deviation for position **H** compared to the other positions. Notably, it is still well below the measured differences in naturally abundant ^2^H at this position when comparing PinOH-1 and PMP-1 with the other two starting materials and products.

The relative standard deviations for the other two positions **Me** and **tBu** are all less than 5%. Compared to position **H**, these two positions hold a higher number of possible equivalent ^2^H nuclei, which produce more intense NMR signals and, correspondingly, lower relative variances. The more intense NMR signals also result in higher SNR (**Me**; ≥40, **tBu**; ≥110), which increases the possibility of sufficient reproducibility. However, the differences in ^2^H abundance at these positions are also smaller compared to position **H**, which requires higher measurement precision to enable differentiation. The low SNR can obviously be improved by optimizing the instrumental settings and by collecting more scans, but longer experimental time constitutes a risk since it can also result in less resolution stemming from the unlocked mode. Alternatively, it is possible to further enhance the sensitivity and analyze a smaller amount of material using an ultrahigh field NMR spectrometer equipped with a dedicated ^2^H probe. However, there is a benefit of using a common NMR spectrometer equipped with an ordinary broadband probe that is normally found in chemistry departments. For instance, the majority of the OPCW-designated laboratories will be able to perform the measurements following our procedure. The specialized instruments are, as of today, not as common.

As can be seen in Figure 4, some positions have a slightly higher ^2^H mean value in the starting material compared to the product (e.g., PinOH-1 and PMP-1 in the **tBu** position), while some positions have a slightly lower mean value (e.g., PinOH-3 and PMP-3 in the **tBu** position). This indicates that there is no systemic enrichment nor systemic depletion of the ^2^H abundance between the starting materials and products caused by our handling of the samples. The data in Figure 4 proves (to our knowledge, for the first time) that the differences measured in the starting material, in large, remain in the product after two reaction steps. Furthermore, the low values of relative standard deviation indicate robustness to differences in sample purity and concentration within our method.

**Figure 5:**
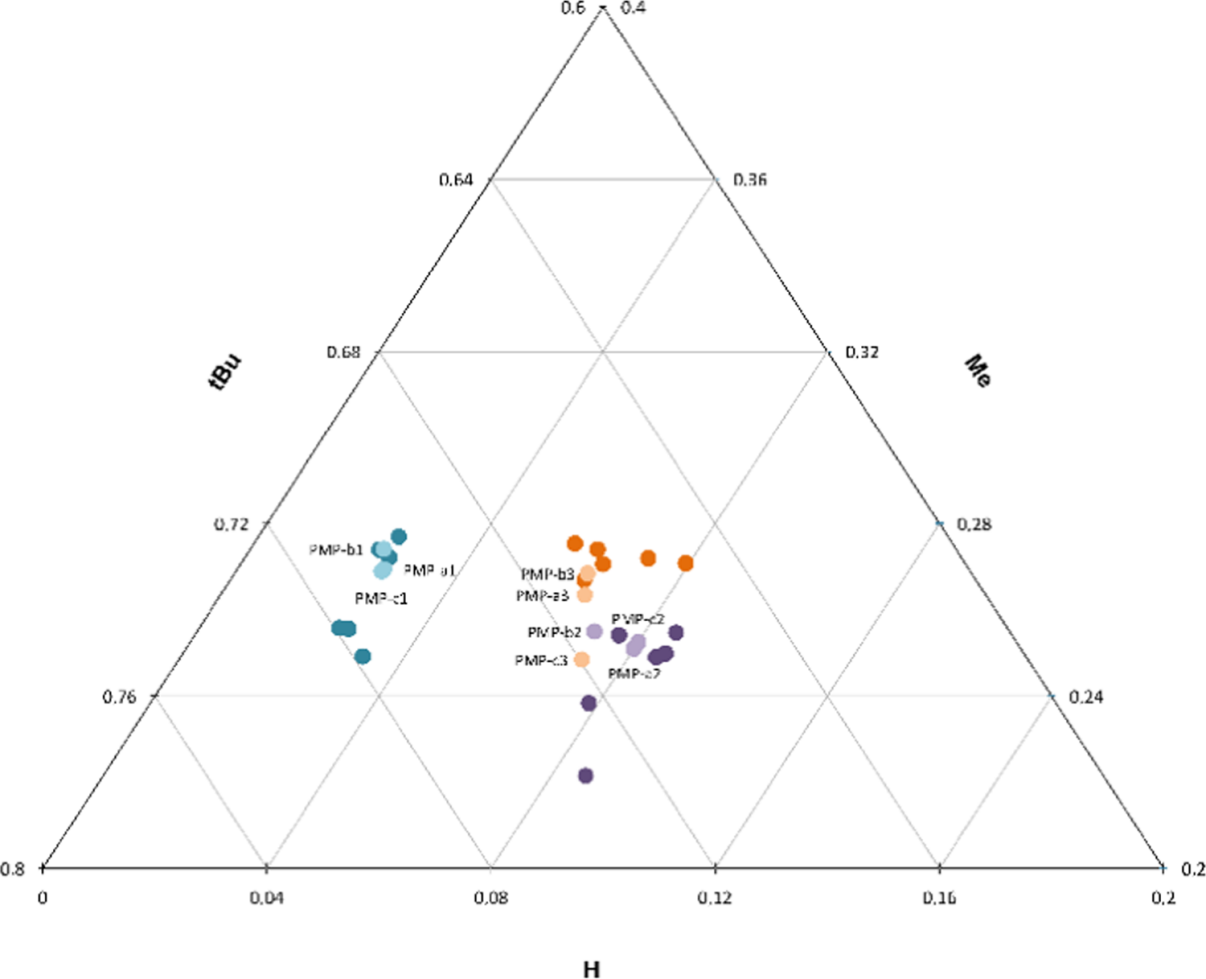
Ternary plot showing all normalized ^2^H peak areas for the precursors (PinOH-1-3_1 (dil., *n* = 3) and PinOH-1-3_2 (neat, *n* = 3), dark) and the products PMP-a1-c3 (*n* = 3, light). The sum of the ^2^H peak areas for all three positions equals one.

To be able to affiliate the precursor PinOH to the product synthesized thereof, the normalized peak area for the precursors together with the products are visualized in a ternary plot (Figure 5). Both concentrations of the starting materials (PinOH-1-3_1 (dil.) and PinOH-1-3_2 (neat), dark colors, *n* = 3) are plotted together with the mean value of all products (PMP-a1-c3, light colors, *n* = 3). Again, the sum of the ^2^H peak areas for all positions equals one.

According to Figure 5, all products are positioned close to their corresponding starting materials. Thus, the natural ^2^H abundance in the product resembles the ^2^H abundance of the precursor. This confirms the conservation of the intramolecular ^2^H fingerprint through synthesis and hydrolysis. When it comes to affiliation, PMP-1 (PMP-a1, PMP-b1, and PMP-c1) has the same unique isotopic profile as PinOH-1 (PinOH-1_1 and PinOH-1_2) in position **H** and is therefore distinctly separated from the other two groups. PMP-2 (PMP-a2, PMP-b2, and PMP-c2) and PMP-3 (PMP-a3, PMP-b3, and PMP-c3) have, as their precursors, a more similar isotopic distribution. However, they are separated in position **Me**, and there is a tendency for two separate clusters. Overall, the majority of the products are positioned close to the correct precursors, except for PMP-c3. The product PMP-c3 has a ^2^H abundance in the **Me** position that resembles the values of PinOH-2 and PMP-2. All three replicates measured for PMP-c3 are accurate but differ from the other two products (PMP-a3 and PMP-b3) in the **Me** position. Within the framework of this study, these differences are not possible to explain without additional data and analysis.

In a real case scenario, where a suspected CWA has been sampled at the scene, it is probably the intact CWA or its hydrolyzed product that is analyzed first. Subsequently, the search for additional proof will be to link the CWA to its precursor. Therefore, it is also required to be able to attribute the precursor to the product. When affiliating the PinOHs (both conc. and dil.) to the PMPs according to Figure 5, all six precursors are grouped to the correct product.

## Conclusions

Our study has shown that PSIA by ^2^H NMR on an alkyl chain of CWAs can be used both to differentiate starting materials and/or PMPs in addition to attributing a nerve agent to its precursor origin. Our results also indicate that the attribution might be possible long after the nerve agent has been dispersed and even after it has been degraded to the corresponding alkyl methylphosphonate.

The different PinOHs in this study have unique intramolecular ^2^H isotopic distributions within the pinacolyl moiety, and this isotopic profile remains through the synthesis of Soman and hydrolysis to PMP. Given our results, our study can be seen as a proof of concept in relation to site-specific isotopic affiliation. Hence, the method has the potential to be a powerful tool that can contribute to forensic investigations in the context of CWA. To make this method more robust, future studies could focus on the construction of statistical prediction models, including blinded samples.

## Supporting Information Available

Representative ^2^H NMR spectra of experimental and simulated data including the residual; the fitted shapes of the deconvolution; and table of all normalized peak areas (PDF)
