# Supplementary material for: Source Attribution of the Chemical Warfare Agent Soman Using Position-Specific Isotope Analysis by 2H NMR Spectroscopy: From Precursor to Degradation Product
Source: Anal Chem. 2021 Sep 1;93(36):12230–6. doi: 10.1021/acs.analchem.1c01271 (PMC8444188; doi:10.1021/acs.analchem.1c01271)
Supplement: Supplementary file 1 — ac1c01271_si_001.pdf [file ac1c01271_si_001.pdf]

Supporting Information

Manuscript Title: Source Attribution of the Chemical Warfare Agent Soman using Position-Specific Isotope Analysis by ^{2}H NMR spectroscopy: From precursor to degradation product

Authors: Sandra Lindberg*, Magnus Engqvist, Lina Mörén, Crister Åstot, Rikard Norlin

Department of CBRN Defence & Security, The Swedish Defence Research Agency (FOI), Cementvägen 20, Umeå, SE-901 82, Sweden
Email: sandra.lindberg@foi.se

Contents: **Page**

The difference between ^{2}H NMR experimental data and deconvolution fit of PinOH-1 (Figure S1) S-2
The deconvolution fitted shapes of PinOH-1 (Figure S2) S-2
The difference between ^{2}H NMR experimental data and deconvolution fit of PinOH-2 (Figure S3) S-3
The deconvolution fitted shapes of PinOH-2 (Figure S4) S-3
Deconvolution peak list of PinOH-2 (Figure S5) S-4
The difference between ^{2}H NMR experimental data and deconvolution fit of PinOH-3 (Figure S6) S-5
The deconvolution fitted shapes of PinOH-3 (Figure S7) S-5
The difference between ^{2}H NMR experimental data and deconvolution fit of PMP-c1 (Figure S8) S-6
The deconvolution fitted shapes of PMP-c1 (Figure S9) S-6
The difference between ^{2}H NMR experimental data and deconvolution fit of PMP-a2 (Figure S10) S-7
The deconvolution fitted shapes of PMP-a2 (Figure S11) S-7
The difference between ^{2}H NMR experimental data and deconvolution fit of PMP-b3 (Figure S12) S-8
The deconvolution fitted shapes of PMP-b3 (Figure S13) S-8
Table of all relative peak areas and estimated uncertainties S-9

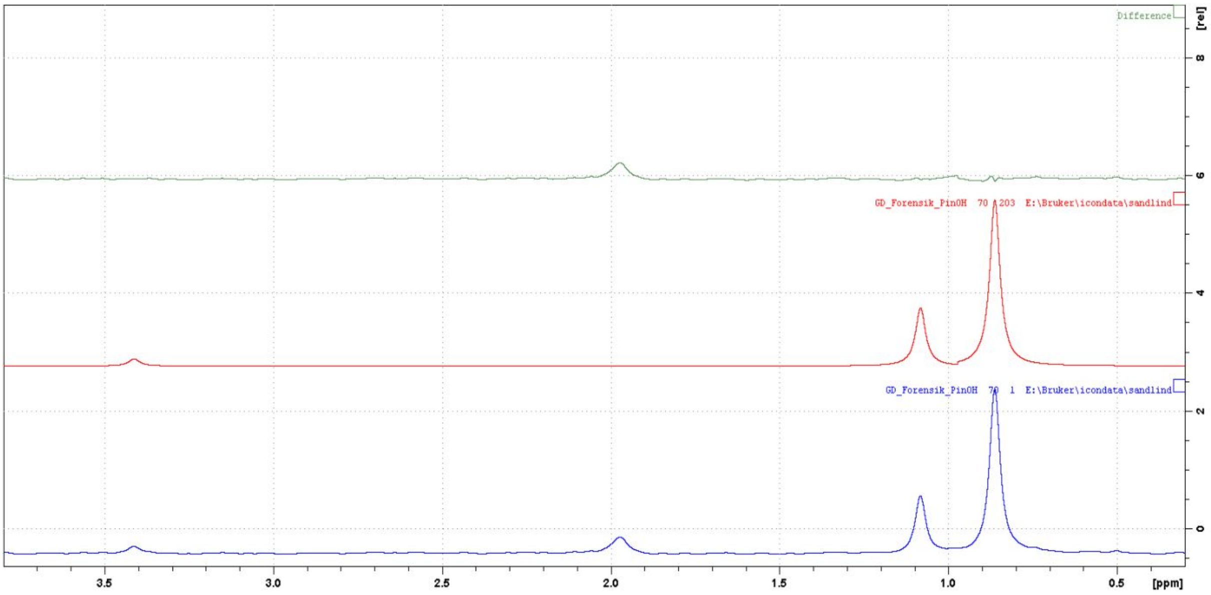

Figure S1. Experimental data of PinOH-1 shown at the bottom (blue), the deconvolution fit shown in the middle (red) and the difference at top (green). The truncations in the deconvolution fit is a graphical error and does not affect the numeric calculations.

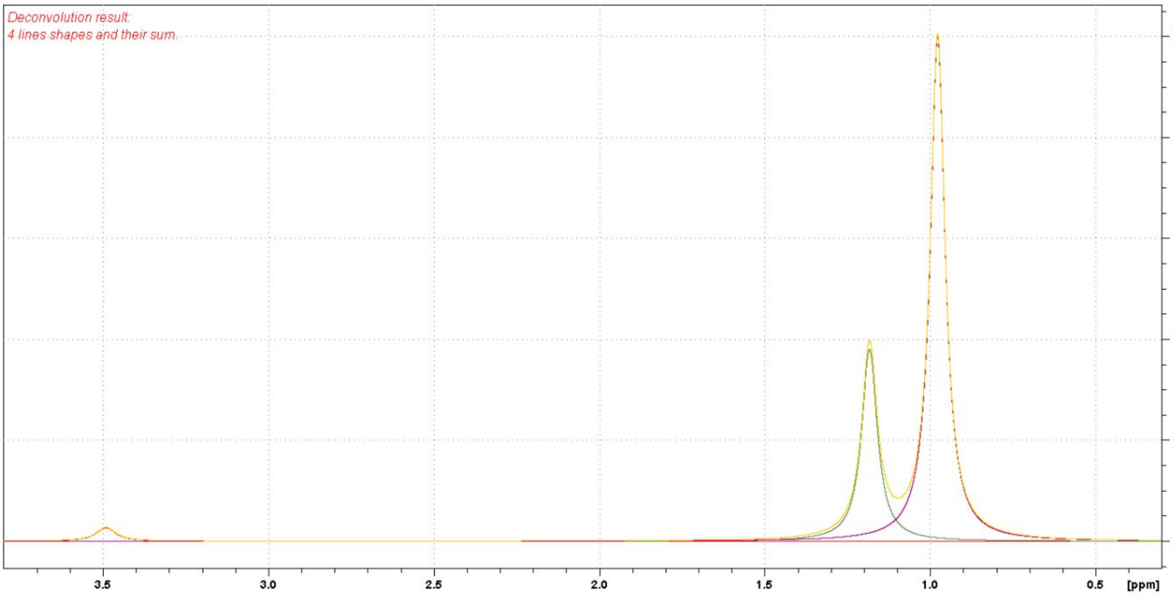

Figure S2. The fitted shapes and calculated area of the deconvolution of PinOH-1. The fit is performed with a 100% Lorenzian lineshape for the different peaks; **H** (red), **Me** (green) and **tBu** (purple).

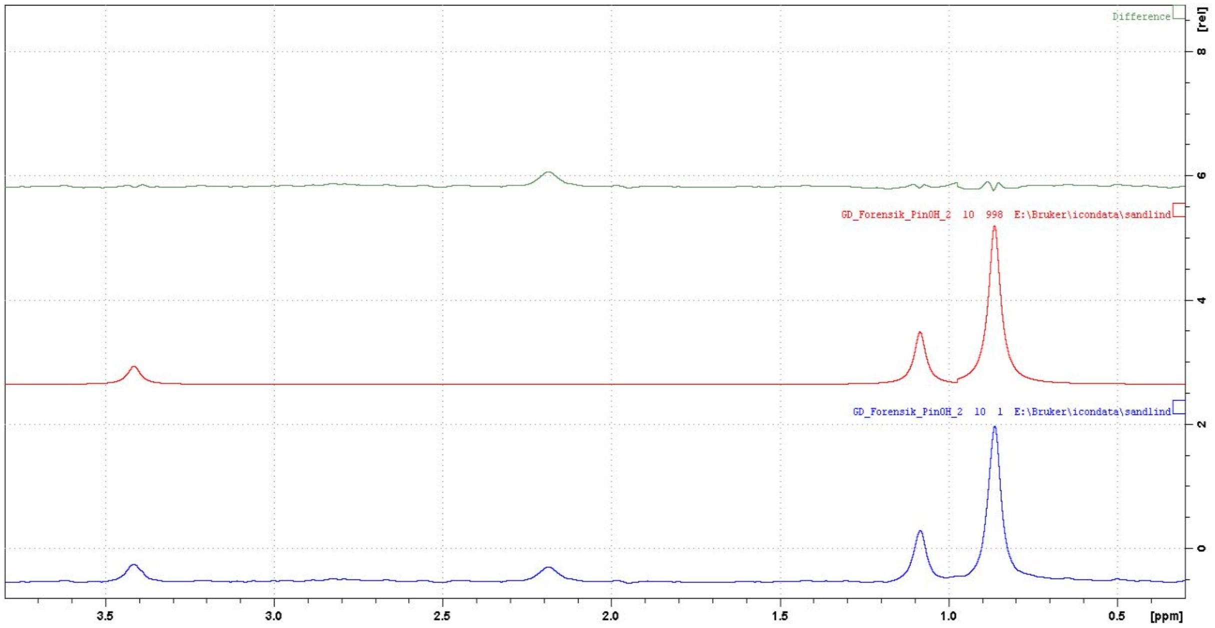

Figure S3. Experimental data of PinOH-2 shown at the bottom (blue), the deconvolution fit shown in the middle (red) and the difference at top (green). The truncations in the deconvolution fit is a graphical error and does not affect the numeric calculations.

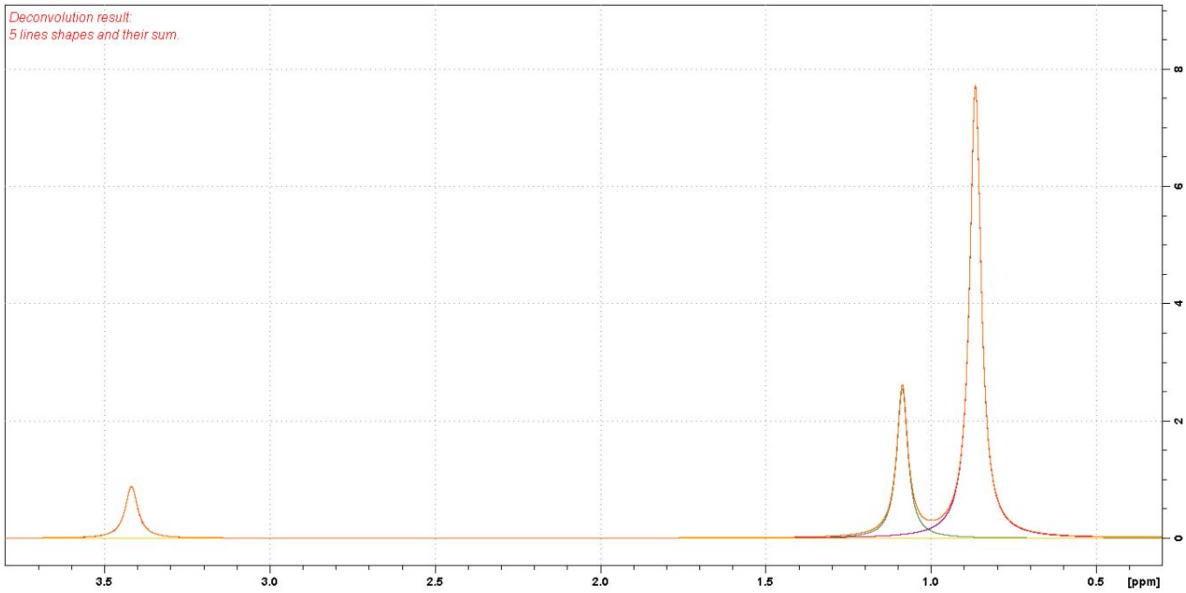

Figure S4. The fitted shapes and calculated area of the deconvolution of PinOH-2. The fit is performed with a 100% Lorenzian lineshape for the different peaks; **H** (red), **Me** (green) and **tBu** (purple).

Tue May 25 11:11:46 CEST 2021

Data set: E:/Bruker/icondata/sandlind/GD_Forensik_PinOH_2/10/pdata/1/

Fit type: Mixed Lorentzian and Gaussian

Fit	Frequency		Width		Intensity	Area	%Lor.
	ppm	Hz	ppm	Hz			chisq
1							1.1e+016
	7.284	559.19	0.03741	2.872	10.270	1977.524	100.00
STD:	0.000	0.00	0.00008	0.006	0.015		
2							2.5e+015
	3.416	262.25	0.04448	3.415	0.883	202.069	100.00
STD:	0.000	0.01	0.00037	0.028	0.005		
3							1.4e+016
	1.085	83.30	0.03963	3.042	2.546	519.162	100.00
STD:	0.000	0.01	0.00045	0.034	0.020		
4							1.8e+015
	0.864	66.35	0.04096	3.144	7.704	1623.949	100.00
STD:	0.000	0.00	0.00008	0.006	0.011		
5							7e+014
	-0.016	-1.25	0.03699	2.840	5.041	959.743	100.00
STD:	0.000	0.00	0.00008	0.006	0.007		

Figure S5. An example of a deconvolution peak list of PinOH-2_1 with numerical data used for normalization of the peak areas; **H** (3.416 ppm), **Me** (1.085 ppm) and **tBu** (0.864 ppm). The CDCl3 (7.284ppm) and the TMS (-0.016ppm) peaks are not considered in this study. The total peak area is 2345.18 and the normalized areas are; **H** = 0.0862, **Me** = 0.221 and **tBu** = 0.692, see Table S1.

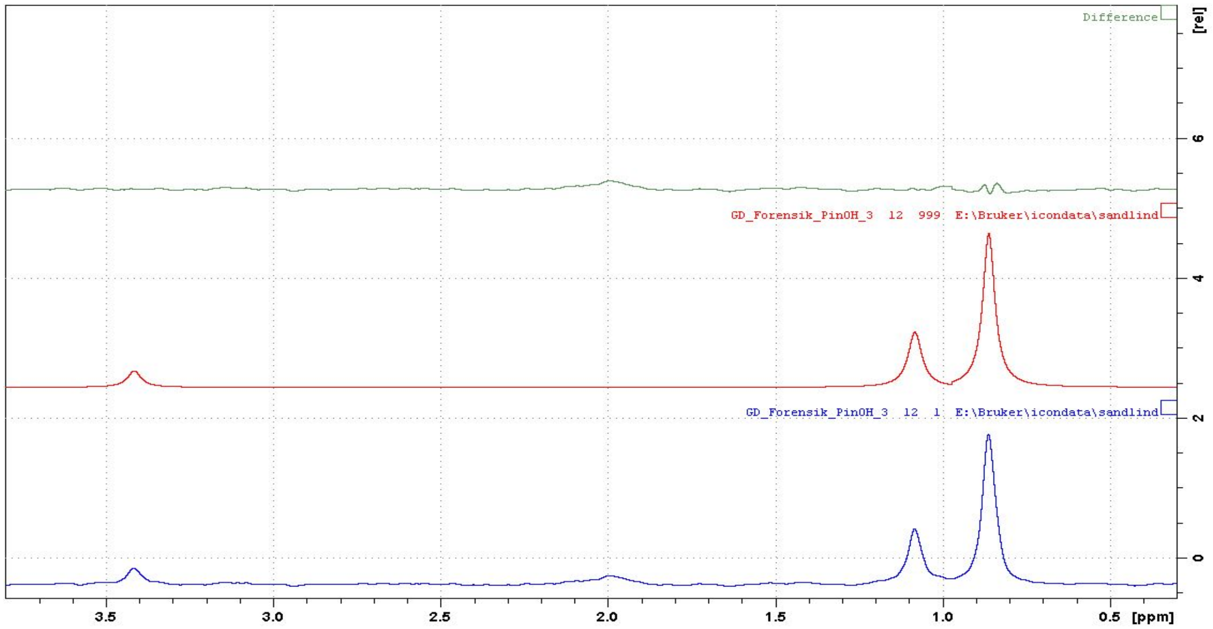

Figure S6. Experimental data of PinOH-3 shown at the bottom (blue), the deconvolution fit shown in the middle (red) and the difference at top (green). The truncations in the deconvolution fit is a graphical error and does not affect the numeric calculations.

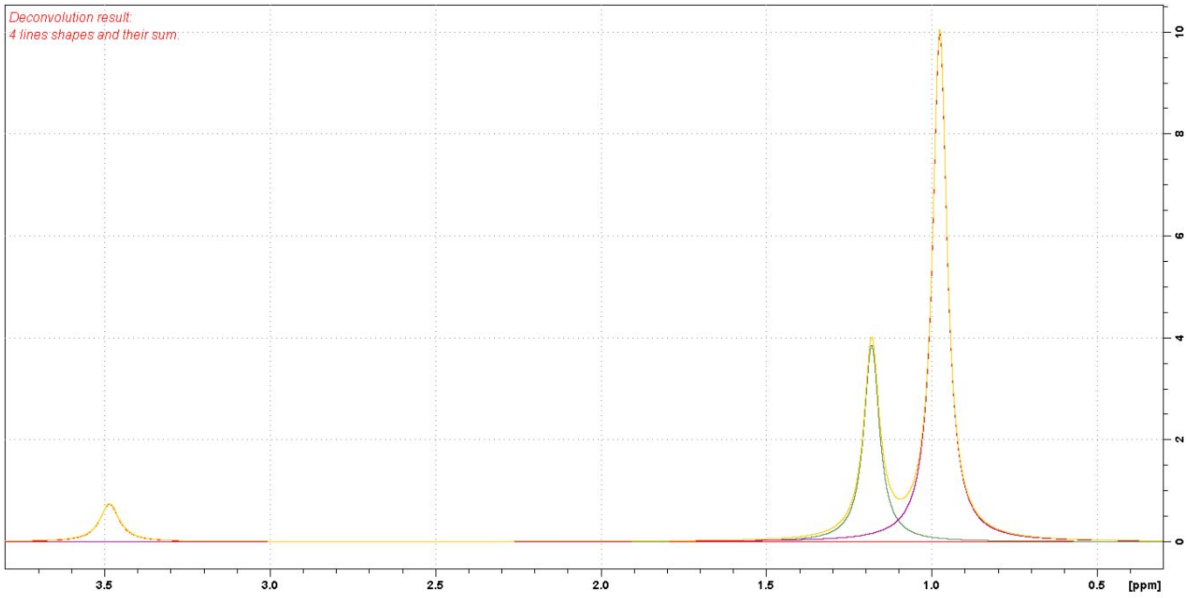

Figure S7. The fitted shapes and calculated area of the deconvolution of PinOH-3. The fit is performed with a 100% Lorenzian lineshape for the different peaks; **H** (red), **Me** (green) and **tBu** (purple).

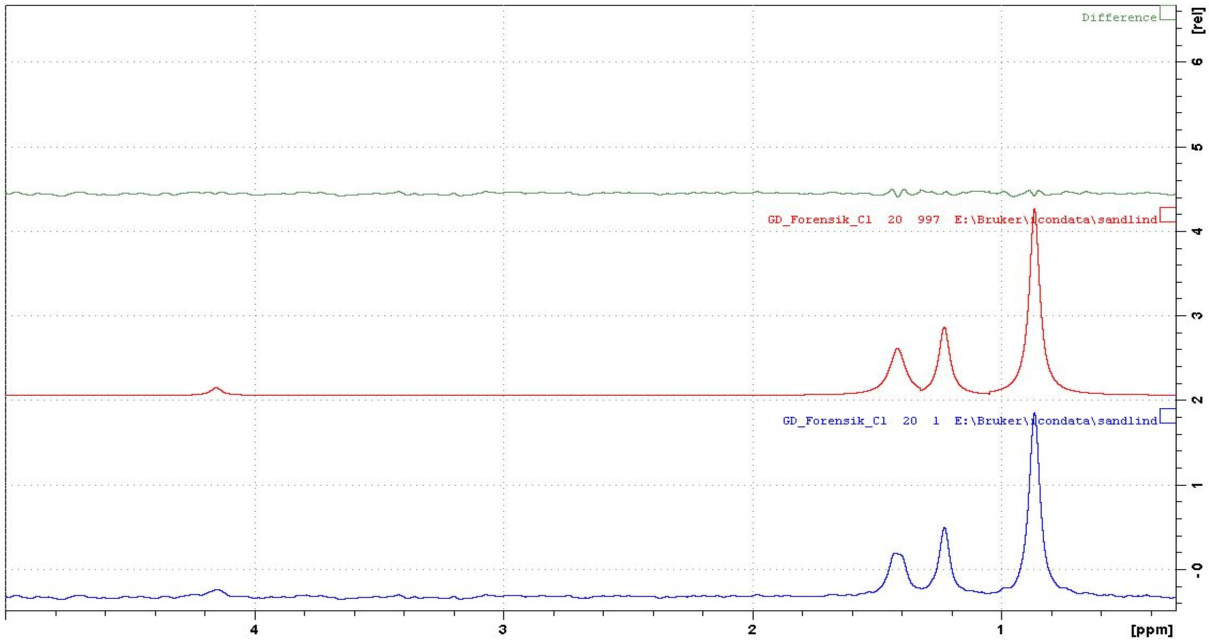

Figure S8. Experimental data of PMP-c1 shown at the bottom (blue), the deconvolution fit shown in the middle (red) and the difference at top (green). The truncations in the deconvolution fit is a graphical error and does not affect the numeric calculations.

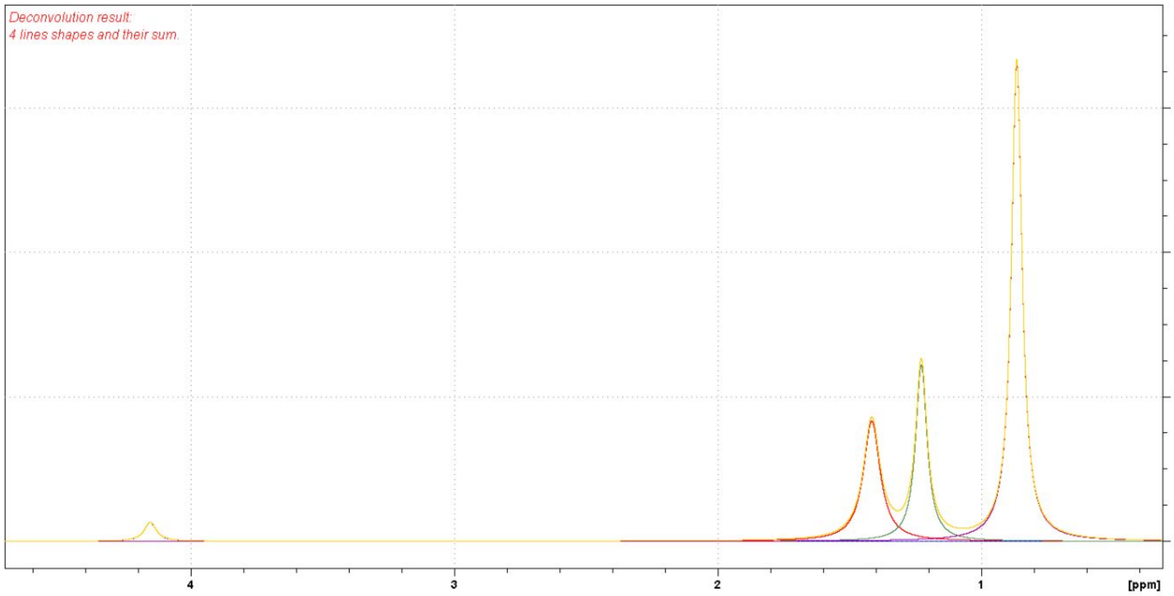

Figure S9. The fitted shapes and calculated area of the deconvolution of PMP-c1. The fit is performed with a 100% Lorenzian lineshape for the different peaks; **H** (blue), **Me** (green) and **tBu** (purple). The **MeP** peak (red) is not considered in this study.

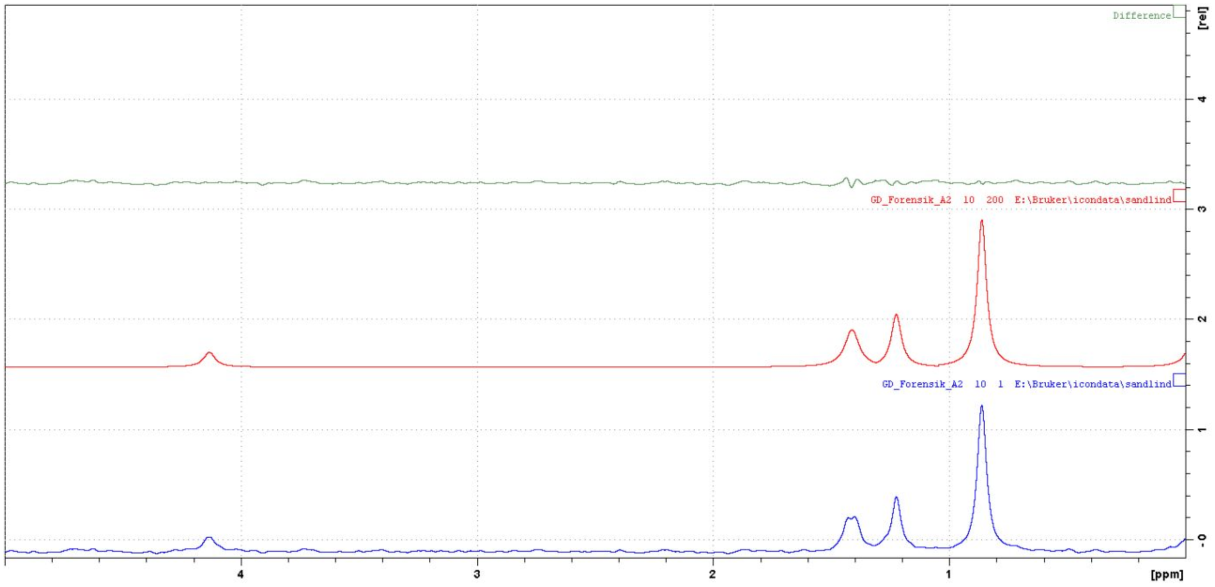

Figure S10. Experimental data of PMP-a2 shown at the bottom (blue), the deconvolution fit shown in the middle (red) and the difference at top (green). The truncations in the deconvolution fit is a graphical error and does not affect the numeric calculations.

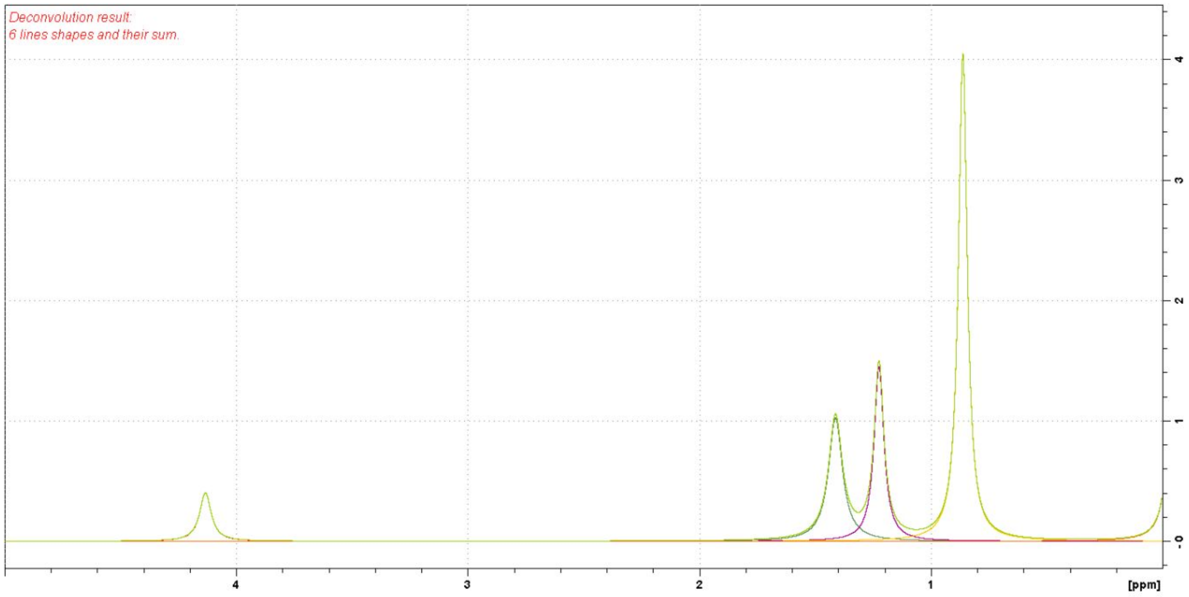

Figure S11. The fitted shapes and calculated area of the deconvolution of PMP-a2. The fit is performed with a 100% Lorenzian lineshape for the different peaks; **H** (light green), **Me** (purple) and **tBu** (yellow). The **MeP** peak (dark green) is not considered in this study.

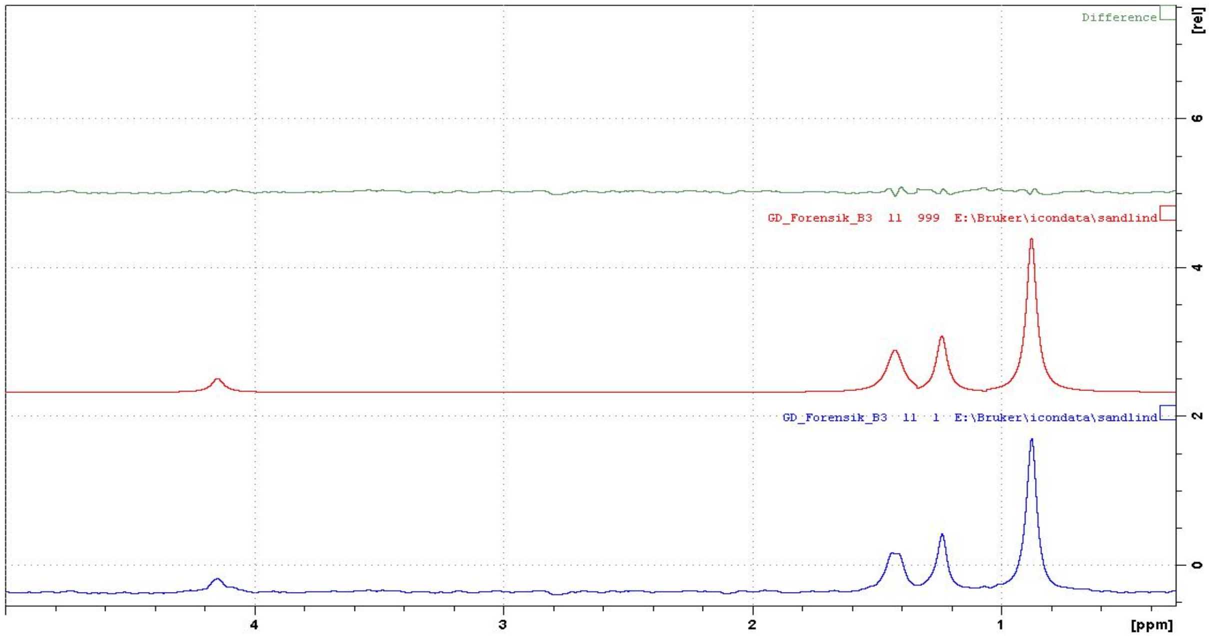

Figure S12. Experimental data of PMP-b3 shown at the bottom (blue), the deconvolution fit shown in the middle (red) and the difference at top (green). The truncations in the deconvolution fit is a graphical error and does not affect the numeric calculations.

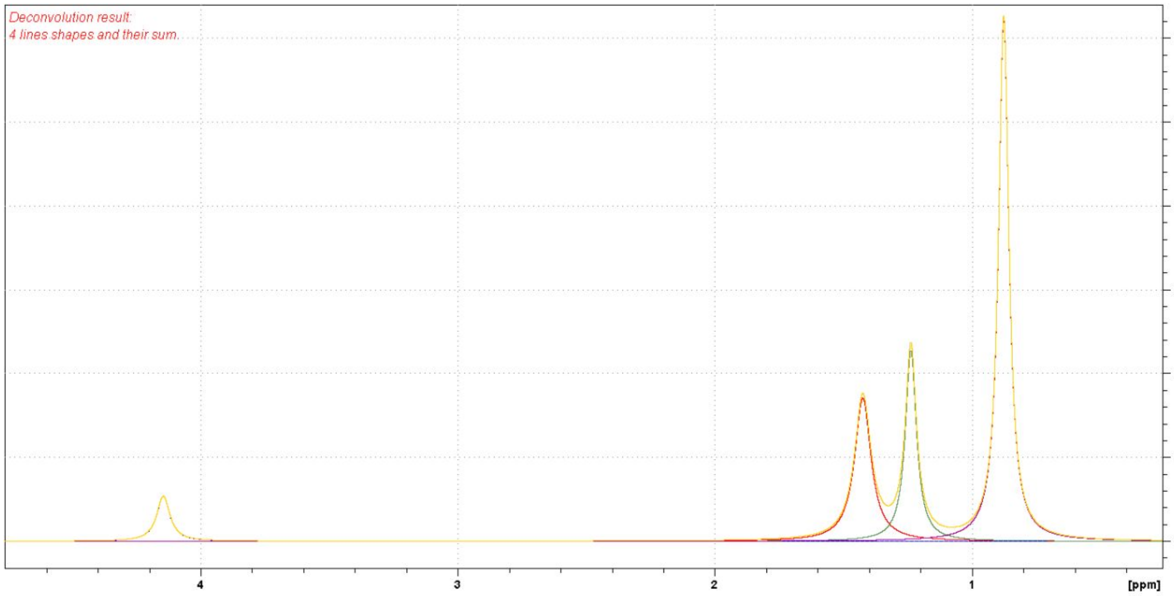

Figure S13. The fitted shapes and calculated area of the deconvolution of PMP-b3. The fit is performed with a 100% Lorenzian lineshape for the different peaks; **H** (blue), **Me** (green) and **tBu** (purple). The **MeP** peak (red) is not considered in this study.

Table S1. Table of relative peak areas for all three replicates with estimated uncertainties (SD).

	PinOH-1_1	PinOH-1_2	PinOH-2_1	PinOH-2_2	PinOH-3_1	PinOH-3_2	PMP-a1	PMP-b1	PMP-c1	PMP-a2	PMP-b2	PMP-c2	PMP-a3	PMP-b3	PMP-c3
H_1	0.0325	0.0258	0.0862	0.0857	0.0721	0.0573	0.0186	0.0216	0.0297	0.0853	0.0739	0.0686	0.0735	0.0638	0.0714
H_2	0.025	0.0231	0.0758	0.0862	0.0632	0.062	0.0247	0.0224	0.023	0.0832	0.068	0.0857	0.0668	0.0709	0.0724
H_3	0.0268	0.0251	0.0783	0.0849	0.0794	0.0647	0.0352	0.0273	0.0243	0.0716	0.0721	0.086	0.0557	0.053	0.0712
Mean	0.0281	0.0247	0.0801	0.0856	0.0716	0.0613	0.0262	0.0238	0.0257	0.0800	0.0713	0.0801	0.0653	0.0626	0.0717
SD	0.00392	0.00140	0.00543	0.000656	0.00811	0.00375	0.00840	0.00309	0.00355	0.00738	0.00302	0.00996	0.00899	0.00901	0.000643

	PinOH-1_1	PinOH-1_2	PinOH-2_1	PinOH-2_2	PinOH-3_1	PinOH-3_2	PMP-a1	PMP-b1	PMP-c1	PMP-a2	PMP-b2	PMP-c2	PMP-a3	PMP-b3	PMP-c3
Me_1	0.249	0.272	0.221	0.255	0.272	0.275	0.281	0.274	0.271	0.263	0.25	0.255	0.261	0.264	0.241
Me_2	0.256	0.274	0.254	0.250	0.267	0.274	0.255	0.274	0.266	0.249	0.257	0.248	0.266	0.262	0.252
Me_3	0.256	0.277	0.238	0.249	0.271	0.271	0.272	0.274	0.27	0.24	0.257	0.253	0.263	0.28	0.253
Mean	0.254	0.274	0.238	0.251	0.27	0.273	0.269	0.274	0.269	0.251	0.255	0.252	0.263	0.269	0.249
SD	0.00404	0.00252	0.0165	0.00322	0.00265	0.00208	0.0132	0	0.00265	0.0116	0.00404	0.00361	0.00252	0.00987	0.00666

	PinOH-1_1	PinOH-1_2	PinOH-2_1	PinOH-2_2	PinOH-3_1	PinOH-3_2	PMP-a1	PMP-b1	PMP-c1	PMP-a2	PMP-b2	PMP-c2	PMP-a3	PMP-b3	PMP-c3
tBu_1	0.718	0.702	0.692	0.660	0.656	0.667	0.7	0.704	0.699	0.652	0.676	0.676	0.665	0.673	0.688
tBu_2	0.719	0.703	0.67	0.664	0.67	0.664	0.72	0.704	0.711	0.668	0.675	0.666	0.667	0.667	0.676
tBu_3	0.718	0.698	0.683	0.666	0.65	0.665	0.693	0.698	0.705	0.688	0.671	0.661	0.682	0.667	0.676
Mean	0.718	0.701	0.682	0.663	0.659	0.665	0.704	0.702	0.705	0.669	0.674	0.668	0.671	0.669	0.68
SD	0.000577	0.00265	0.0111	0.00306	0.0103	0.00153	0.0140	0.00346	0.006	0.0180	0.00265	0.00764	0.00930	0.00346	0.00693
